# Mortality, morbidity, and cardiac surgery in Injection Drug Use (IDU)-associated versus non-IDU infective endocarditis: The need to expand substance use disorder treatment and harm reduction services

**DOI:** 10.1371/journal.pone.0225460

**Published:** 2019-11-26

**Authors:** Kinna Thakarar, Kristina E. Rokas, F. L. Lucas, Spencer Powers, Elizabeth Andrews, Christina DeMatteo, Deirdre Mooney, Marcella H. Sorg, August Valenti, Mylan Cohen

**Affiliations:** 1 Center for Outcomes Research and Evaluation, Maine Medical Center Research Institute, Portland, ME, United States of America; 2 Tufts University School of Medicine, Boston, MA, United States of America; 3 Maine Medical Center, Portland, ME, United States of America; 4 InterMed Infectious Disease, South Portland, ME, United States of America; 5 Margaret Chase Smith Policy Center, University of Maine, Orono, ME, United States of America; Columbia University, UNITED STATES

## Abstract

**Background:**

The addiction crisis is widespread, and unsafe injection practices among people who inject drugs (PWID) can lead to infective endocarditis.

**Methods:**

A retrospective analysis of adult patients with definite or possible infective endocarditis admitted to a tertiary care center in Portland, Maine was performed over three-year period. Our primary objective was to examine differences in demographics, health characteristics, and health service utilization between injection drug use (IDU)-associated infective endocarditis and non-IDU infective endocarditis. The association between IDU and mortality, morbidity (defined as emergency department visits within 3 months of discharge), and cardiac surgery was examined. Bivariate and multivariate analyses were performed. A subgroup descriptive analysis of PWID was also performed to better examine substance use disorder (SUD) characteristics, treatment with medication for opioid use disorder (MOUD) and health service utilization.

**Results:**

One-hundred and seven patients were included in the study, of which 39.2% (n = 42) had IDU-associated infective endocarditis. PWID were more likely to be homeless, uninsured, and lack a primary care provider. PWID were notably younger and had less documented comorbidities, however had similar in-hospital mortality rates (10% vs. 14%, p = 0.30), ED visits (50% vs. 54%, p = 0.70) and cardiac surgery (33% vs. 26%, p = 0.42) compared to those with non-IDU infective endocarditis. Ninety-day mortality was less among PWID (19.0% vs. 36.9%, p = 0.05). IDU was not associated with morbidity (adjusted odds ratio (AOR) 0.73, 95% CI 0.18–3.36), 90-day mortality (AOR 0.72, 95% CI 0.17–3.01), or cardiac surgery (AOR 0.15, 95% CI 0.03–0.69). Ninety-day mortality among PWID who received MOUD was lower (3% vs 15%, p = 0.45), as were ED visits (10% vs. 41%, p = 0.42) compared to those who did not receive MOUD.

**Conclusions:**

Our results highlight existing differences in health characteristics and social determinants of health in people with IDU-associated versus non-IDU infective endocarditis. PWID had less comorbidities and were significantly younger than those with non-IDU infective endocarditis and yet still had similar rates of cardiac surgery, ED visits, and in-hospital mortality. These findings emphasize the need to deliver comprehensive health services, particularly MOUD and other harm reduction services, to this marginalized population.

## Background

Unsafe injection practices among people who inject drugs (PWID) can lead to overdoses, skin and soft tissue infections, human immunodeficiency virus (HIV), viral hepatitis and other complications such as infective endocarditis. These complications can result in costly hospitalizations, emergency department (ED) visits, and death [1, 2]. Injection drug use (IDU) is a known risk factor for infective endocarditis [3]. Nationally, hospitalizations from IDU-associated infective endocarditis increased from 7% to 12% between 2000–2013, with recent cases of IDU-associated infective endocarditis shifting to reflect younger PWID who are more likely to be white and female than previously reported [4].

In the United States, the incidence of reportable IDU-associated infections such as acute hepatitis C (HCV) is increasing particularly among rural states[5]. In Maine, where twelve of sixteen counties are considered rural per Health Resources and Services Administration criteria, the impact of infective endocarditis in the state is unknown. [6] While data are lacking on precise numbers of PWID in Maine, the impact of opioid use is evident through data on statewide substance use treatment admissions, ED visits, and overdose deaths.[7] [8].

Reportable IDU-related infectious diseases in Maine have increased dramatically over the past several years, with rates of acute HCV increasing by 20% and acute hepatitis B (HBV) increasing by nearly 500% from 2015 to 2016 [9]. Due to the lack of requirement to report, there is an absence of data about infective endocarditis trends in Maine. In response to the perceived surge of infective endocarditis cases in the setting of the drug use epidemic, we sought to examine differences in demographics, health characteristics, and health service utilization between those with IDU-associated infective endocarditis versus those with non-IDU infective endocarditis. Our study also contributes to existing literature by examining substance use disorder characteristics, particularly use of medication for opioid use disorder (MOUD), among PWID with infective endocarditis.

## Methods

### Study design

A single-center, retrospective analysis was conducted among adult patients with definite or possible infective endocarditis. Data were captured using chart review of the hospital electronic health record (EHR) and HealthInfoNet, a statewide database linking select identifiable medical information (i.e. emergency department visits, mortality, microbiology) from twenty-nine hospitals throughout Maine. The latter was used to supplement EHR data for follow-up variables of interest. HealthInfoNet is opt-in for general medical information and opt-out for behavioral health/HIV status, though less than 1% of patients opt-out of the database,[10]. This study was approved by the institutional review board of Maine Medical Center. Maine Medical Center is a 637-bed academic, tertiary care medical center located in Portland, Maine, and the largest hospital in the state. Patients who received a transesophageal echocardiogram (TEE) for investigation of infective endocarditis from January 1, 2013 and January 1, 2016 were evaluated. The TEE report was extracted from the hospital's cardiovascular database using a query with the indication "endocarditis," and study conclusion including either "endocarditis" or "vegetation." Through chart review, participants were then categorized to definite, possible, or no endocarditis. Only index admissions for definite or possible endocarditis were included in the study. The primary objective was to examine differences in demographics, health characteristics, and health service utilization between those with IDU-associated infective endocarditis versus those with non-IDU infective endocarditis. The associations between IDU and mortality, morbidity, and cardiac surgery were also evaluated. A subgroup descriptive analysis of PWID was performed to better examine substance use disorder (SUD) history, SUD health service utilization, and overdoses.

### Definitions

*“*Definite or possible endocarditis” was determined using the modified Duke criteria[11, 12]. “Mortality" was defined as patient deaths occurring during infective endocarditis admission or within 3 months afterwards and captured using EHR, HealthInfoNet, and medical examiner data. "In-hospital mortality" was defined as death during the infective endocarditis admission. "Morbidity" was defined as emergency department (ED) visits within 3 months post-discharge from the infective endocarditis admission and was captured using EHR and the HealthInfoNet database. "Cardiac surgery" was defined as any patient receiving cardiac surgery within 3 months, including valve repair and replacement, as a result of infective endocarditis.

Urban versus (vs.) rural status was determined using a classification scheme based on the Office of Management and Budget labeling of metro and non-metro areas, respectively[13]. "Homeless" was defined as documentation of living on street, shelter, transitional housing, staying with friends, or documentation of homelessness in EHR review.

Comorbidities during index admission, medications prescribed before and after admission, and referrals before/after admission were collected through EHR review. "Injection drug use" was defined as any self-reported history (i.e. reported in subjective sections of notes), documented history (problem list/past medical history/other medical notes) or documented clinical suspicion of injection drug use in the EHR (i.e. source of infection documented as likely IDU based on physical exam findings or previous history). "Mental health condition" included anxiety, depression, schizophrenia, ADHD, history of a suicide attempt, posttraumatic stress disorder, bipolar and personality disorders. *"*Alcohol use disorder" was categorized as mild, moderate or severe using DSM 5 criteria.” Type of drug injected" was captured using EHR review. "Illicit drugs used" were captured using EHR and toxicology screens (UDS). UDS were considered positive if substances did not appear on the participant's pre-admission medication list or medication administration record. "Days of positive blood culture" was defined using the number of days from the first positive blood culture until the date of the first negative blood culture. Prognostic comorbidity was calculated using the Charlson Comorbidity Index (CCI), which utilizes a weighted score of seventeen comorbidities to help predict long term mortality[14].

"Medication for opioid use disorder" (MOUD) was defined as documentation of buprenorphine, buprenorphine/naloxone, naltrexone, or methadone in the EHR. "Non-fatal overdoses" within 3 months of TEE admission were captured using the EHR and HealthInfoNet data. Intensive care unit (ICU) stays, disposition, length of stays (LOS) were collected using the EHR.

## Statistical analysis

Comparisons between groups were analyzed using Fisher’s exact or chi-square test for categorical data. Continuous variables were compared using Mann-Whitney U test or Student’s t-test, depending on distribution of the data. Bivariate and multivariate analyses using logistic regression were performed to examine the association between IDU and the outcomes of interest. Age, gender, and IDU were included as variables in each regression model. Based on knowledge from prior literature, other potentially confounding variables were also included in the multivariable models if they were statistically significant in the unadjusted odds (bivariate) analyses. Statistical significance was established at the p<0.05 level.

## Results

### Patient selection

**Fig 1:**
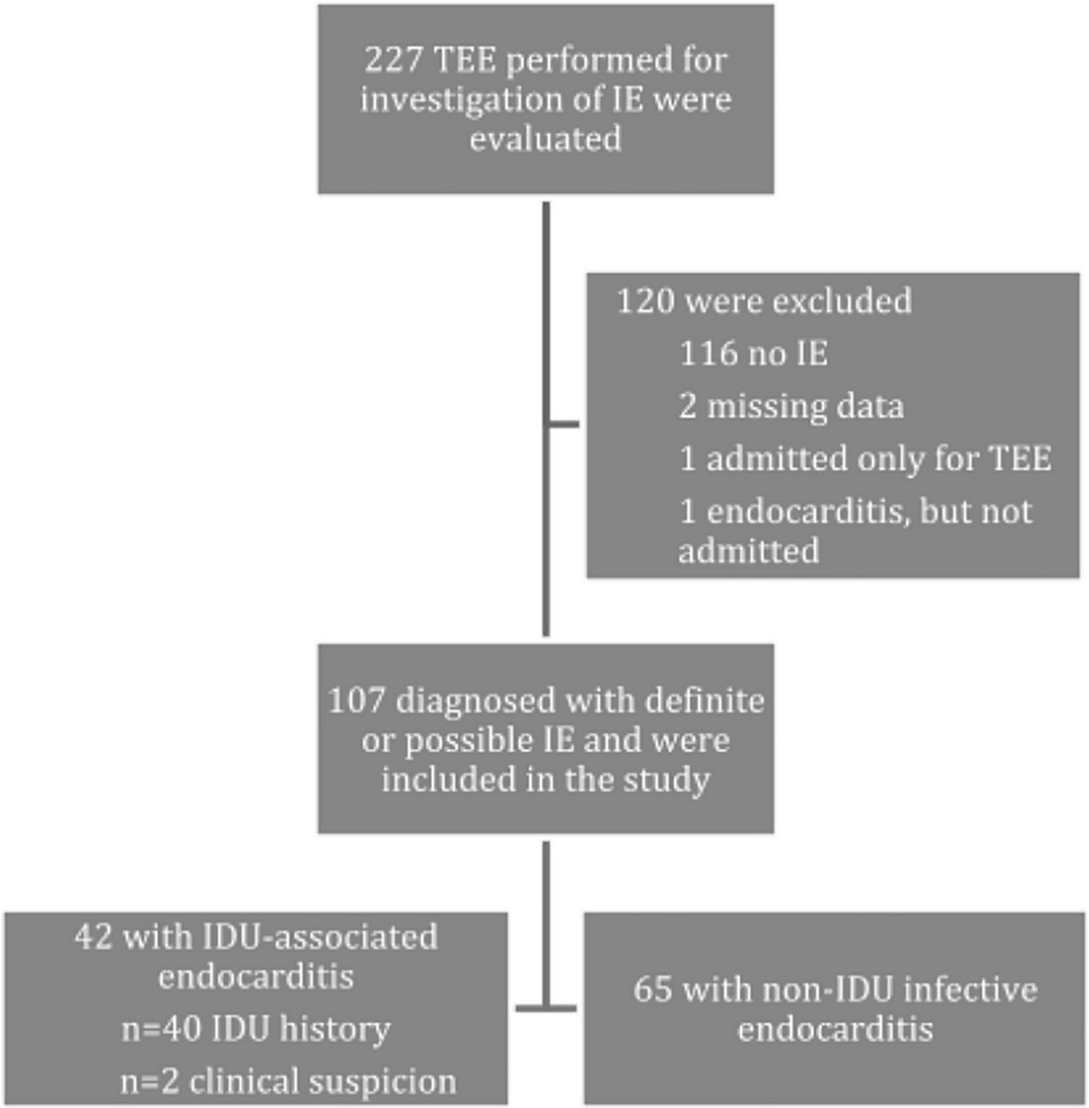
Patient selection: Patients who received transesophageal echocardiography (TEE) for investigation of injection drug use (IDU)-associated infective endocarditis (IE).

**Fig 2:**
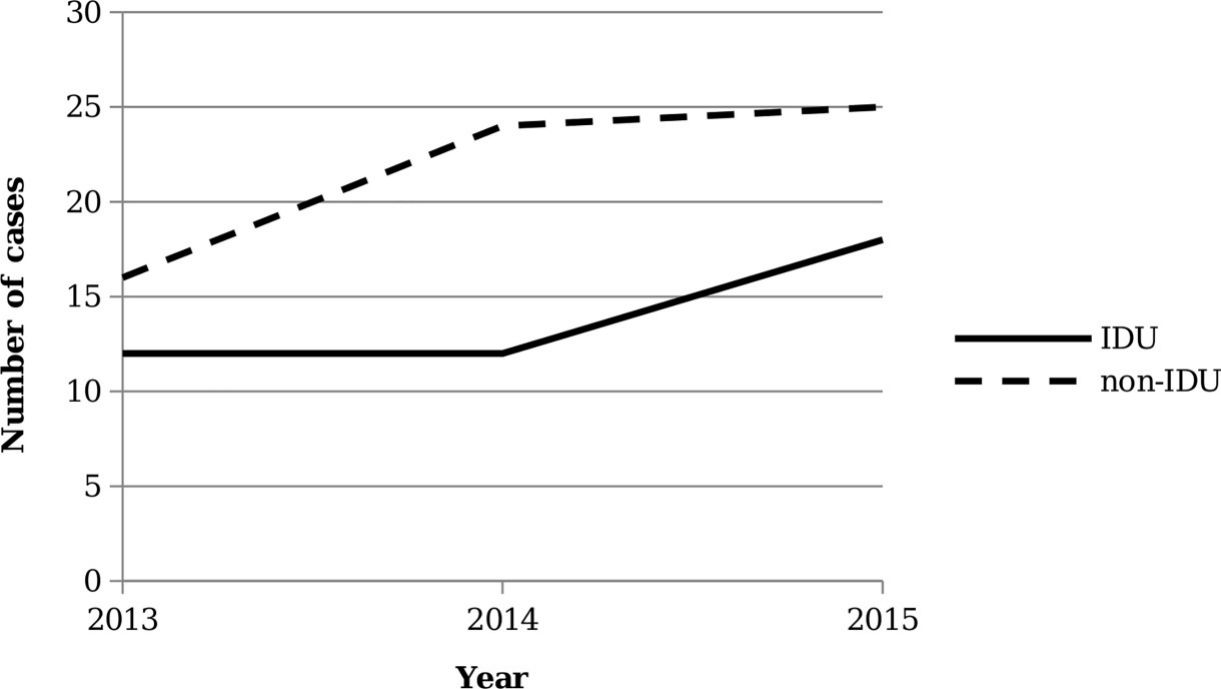
Number of cases of people with IDU-associated infective endocarditis versus non-IDU infective endocarditis.

Of the 227 patients with a TEE performed for infective endocarditis investigation, 107 patients (47.1%) were diagnosed with definite or possible infective endocarditis and included in the study (Fig 1). Forty-two (39%) patients were included in the PWID group. Forty of 42 PWID self-reported IDU and 2 PWID were documented with clinical suspicion of IDU. The overall number of infective endocarditis cases increased by 53.6% during the three-year study period, with a noted 25% increase in the PWID group (Fig 2).

### Demographics

PWID were more likely to be younger (median age 34.8 vs. 63.1 years old, p = 0.0003), have state insurance (71.4% vs. 53.8%) or be uninsured (10.3% vs. 3.1%; p<0.0001), lack a primary care provider (89.2% vs. 71.4%, p = 0.02), or to be homeless (21.4% vs. 1.5%, p = 0.001) compared to those with non-IDU infective endocarditis. **(Table 1).**

**Table 1:** Select demographics and health characteristics, comparing people with IDU-associated infective endocarditis versus people with non-IDU infective endocarditis.

Variable	Total	IDU-IE	Non-IDU-IE	p- value
	n = 107	n = 42	n = 65	
Age, median (25th, 75th))	52.0 (35, 69)	33.5 (27–42)	65 (54–74)	0.003
Male gender	74 (69%)	27 (64%)	47 (72%)	0.38
Insurance^a^				
Private only	19 (18%)	1 (2%)	18 (28%)	<0.001
State only	65 (61%)	30 (71%)	25 (54%)	
Private plus state	10 (9%)	0	10 (15%)	
Uninsured	13 (12%)	11 (26%)	2 (3%)	
Documented PCP	88 (82%)	30 (71%)	58 (89%)	0.02
Race				
White	103 (96%)	41 (98%)	62 (95%)	0.73
African American	1 (1%)	0 (0)	1 (2%)	
Unknown/Other	3 (3%)	1 (2%)	2 (3%)	
Homeless	10 (9%)	9 (21%)	1 (2%)	0.001
From rural location	30 (28%)	11 (26%)	19 (29%)	0.73
Charlson Comorbidity Index^b^				<0.001
Less than 3	48 (45%)	34 (81%)	14 (22%)	
≥ 3	59 (55%)	8 (19%)	51 (78%)	
Hepatitis C				
Yes	32 (17%)	31 (74%)	1 (2%)	<0.001
No	57 (53%)	6 (14%)	51 (78%)	
Not checked	18 (30%)	5 (12%)	13 (20%)	
Mental Health Condition	61 (57%)	42(100%)	19 (29%)	<0.001
Alcohol Use Disorder ^c^	17 (16%)	14 (33%)	3 (5%)	<0.001
**Endocarditis characteristics**				
Definite endocarditis^d^	72 (69%)	36 (88%)	36 (56%)	<0.001
Valve type ^e^				
Right-sided	12 (11%)	9 (21%)	3 (5%)	0.01
Left-sided/left and right-sided	69 (64%)	27 (64%)	42 (65%)	
Type of endocarditis				
Prosthetic	34 (32%)	9 (21%)	25 (39%)	0.07
Native	73 (68%)	33 (79%)	40 (61%)	
Cardiac device	10 (9.4)	1 (2.4)	9 (13.8)	0.05
Pathogen^f^				
Gram-positive	91 (85%)	39 (93%)	52 (80%)	0.57
Staphylococcus aureus	47 (45%)	23 (55%)	25 (39%)	
Methicillin-sensitive	24 (72%)	17 (74%)	17 (68%)	
Methicillin-resistant	13 (28%)	6 (26%)	7 (28%)	
Streptococcus spp.	24 (22%)	8 (19%)	16 (25%)	
Enterococcus spp.	9 (8%)	6 (14%)	3 (5%)	
CoNS	7 (7%)	1 (2%)	6 (9%)	
Other	3 (3%)	1 (2%)	2 (3%)	
Gram-negative	6 (6%)	3 (7%)	3 (5%)	
Yeast	3 (3%)	0 (0%)	3 (5%)	
Vegetation size^g^				
≥ 1cm	52 (48%)	27 (64%)	25 (39%)	0.02
< 1cm	18 (17%)	5 (12%)	13 (20%)	
Vascular phenomena^h^	39 (36%)	24 (57%)	15 (23%)	<0.001
Immunological phenomena^i^	6 (6%)	2 (5%)	4 (6%)	<0.001
Infectious complications^j^	63 (59%)	32 (76%)	31 (48%)	0.003
In-hospital mortality	13 (12%)	4 (10%)	9 (14%)	0.3
90-day mortality	32 (30%)	8 (19%)	24 (37%)	0.05

Data reported in n (%) unless otherwise indicated

Abbreviations: IDU-IE, injection drug use-associated infective endocarditis; non-IDU IE, non-injection drug use associated infective endocarditis; PCP, primary care provider; CoNS, coagulase negative staphylococci; cm = centimeter

^a^ Percentages do not sum to 100% due to rounding

^b^Median of total population was 3

^c^ alcohol use (not otherwise specified) also noted in n = 7 with IDU-IE and n = 2 non-IDU-IE

^d^Total population n = 105, IDU-IE n = 41, non-IDU IE n = 64

^e^ n = 26 valve data not available

^f^Patients could have had an infection with more than one pathogen; susceptibility data not available for n = 1 participant with non-IDU IE

^g^Total population n = 70, valve data not available for n = 37 participants (n = 10 IDU IE, n = 27 non-IDU IE)

^h^Vascular phenomena defined as major arterial emboli, septic pulmonary infarcts, mycotic aneurysm, intracranial hemorrhage, conjunctival hemorrhages, and/or Janeway lesions

^i^Immunological phenomena defined as glomerulonephritis, Osler nodes, Roth spots, and rheumatoid factor

^j^ Infectious complications defined as septic emboli, septic joint, osteomyelitis, skin/soft tissue infection, epidural abscess, or other

### Health characteristics

PWID were more likely to have hepatitis C (73.8% vs. 1.5%, p<0.001), mental health condition (100.0% vs. 29.2%, p<0.001), have a history of cocaine use (38.5% vs. 1.5%, p<0.001), cannabis use (26.2% vs. 6.2%, p = 0.008), amphetamine use (9.5% vs. 0.0%, p = 0.02), any illicit (non-injection) drug use (66.7% vs. 7.7%, p<0.001), and alcohol use disorder (33.3% vs. 4.6%, p<0.001) compared to those with non-IDU infective endocarditis. People with non-IDU infective endocarditis were more likely to have a higher Charlson Index comorbidity score (78.4% vs. 19%, p<0.001) **(Table 1)**. No patients had a history of HIV. All 44 patients (41.1%) tested for HIV during their hospitalization had negative results.

### Endocarditis characteristics

PWID were more likely to have vegetations >1 cm (64.3% vs. 38.5% p = 0.02), vascular phenomena (57.1% vs. 38.5%, p = <0.001) and other infectious complications (76.2% vs. 47.7%, p = 0.003) compared to those with non-IDU infective endocarditis. There were also more cases of right-sided infective endocarditis among the IDU population (21.4% vs. 4.6%, p = 0.01). Participants with IDU-associated infective endocarditis were less likely to have prosthetic valve endocarditis (21.8% vs. 38.7%, p = 0.07), There were no statistically significant differences between IDU infective endocarditis and non-IDU infective endocarditis in history of endocarditis (14.1% vs. 10.8%, p = 59), conduction abnormality (30.9% vs. 27.7%, p = 0.80), undergoing transthoracic echocardiogram (78.6 vs. 75.4%, p = 0.70), persistent blood cultures beyond 3 days (29.4% vs. 56%, p = .10), or receiving empirical antibiotics within approximately 24 hours (9.5% vs. 12.3% p = 0.41). Similar to those with non-IDU infective endocarditis., the most commonly implicated pathogen among those with IDU-associated infective endocarditis was *Staphylococcus aureus* (54.8%) followed by *Streptococcus* spp (22.4%).

### Morbidity and mortality

Ninety-day mortality was less among PWID (19.0% vs. 36.9%, p = 0.05) although this difference was of borderline statistical significance. In-hospital mortality was similar among both groups (10% vs. 14%, p = 0.30). There were no statistically significant differences in ED visits within 3 months of admission (50.0% vs. 53.8%, p = 0.70) or cardiac surgery (33.3% vs. 26.2%, p = 0.42) in PWID vs. those with non-IDU infective endocarditis.

### Health service utilization

PWID were more likely to remain hospitalized for intravenous (IV) antibiotic therapy (35.7% vs. 4.6%, p<0.001), and have a psychiatry consult (76.6% vs. 15.4%, p<0.001) compared to those with non-IDU infective endocarditis. There were no statistically significant differences in 30-day re-admissions (21.4% vs. 26.2%, p = 0.58), receipt of infectious disease consultative services (97.6% vs. 98.5%, p = 0.75), cardiology consultative services (61.9% vs. 70.8%, p = 0.34), and cardiothoracic (CT) surgery consultative services (64.3 vs. 55.4%, p = 0.36) among PWID vs. those with non-IDU infective endocarditis. **(Table 2)**.

**Table 2:** Health service utilization in patients with infective endocarditis, comparing people with IDU-associated infective endocarditis versus people with non-IDU infective endocarditis.

Variable	Total	IDU-IE	Non-IDU IE	p value
	n = 107	n = 42	n = 65	
Length of stay^a^				
< 14 days	58 (54%)	18 (43%)	40 (62%)	
≥ 14 days	49 (46%)	24 (57%)	25 (38%)	0.06
ICU admission	67 (63%)	30 (71%)	37 (57%)	0.13
ED visit ≤ 3 months of discharge	56 (52%)	21 (50%)	35 (54%)	0.7
30-day re-admission	26 (24%)	9 (21%)	17 (26%)	0.58
Disposition				
In-hospital death	13 (12%)	4 (10%)	9 (14%)	<0.001
Home with PICC	39 (36%)	10 (24%)	29 (45%)	
Home, to infusion center	4 (4%)	3 (7%)	1 (2%)	
Discharged to facility	19 (18%)	3 (7%)	16 (25%)	
Inpatient only	18 (17%)	15 (36%)	3 (5%)	
Left prior to therapy completion	5 (5%)	5 (12%)	0 (0)	
Hospice	1 (1%)	0 (0.0)	1 (12%)	
Unknown^b^	9 (8%)	3 (7%)	6 (9%)	
Cardiac surgery	31 (29%)	14 (33%)	17 (26%)	0.42
ID consultation	105 (98%)	42 (100%)	63 (97%)	0.52

Data reported in n (%) unless otherwise indicated

Abbreviations: IDU-IE, injection drug use-associated infective endocarditis; non-IDU IE, non-injection drug use associated infective endocarditis; ICU, intensive care; ED, emergency department; PICC, peripherally inserted central catheter; IV, intravenous; ID, infectious diseases

^a^Median length of stay of total population was 14 days

^b^3 participants with IDU-IE and n = 6 with non-IDU IE were discharged with a PICC line to an unspecified location. Percentages do not sum to 100% due to rounding

### Subgroup analyses of PWID

#### Substance use disorder history

Among PWID, heroin was most commonly reported as the type of drug injected (59.5%), followed by unspecified opioid (26.2%), cocaine (19.0%), buprenorphine/naloxone (16.7%), other drug (9.5%), unknown (14.3%), oxycodone (7.1%), and buprenorphine (2.4%). Most PWID reported injecting drugs for a six month or longer duration prior to admission (69%). Fentanyl was not self-reported by any study participants, and is also not available on local urine toxicology screens. Among PWID who reported other illicit, non-injection drug use, cocaine use (38.1%) was most commonly reported. Amphetamine use (9.5%), cannabis use (26.2%), and alcohol use disorder (33%) were also prevalent among PWID.

#### Health service utilization

Prior to admission, 11.9% of PWID were prescribed MOUD. Upon discharge, 23.8% were discharged on MOUD. Notably, 38.1% were discharged on a prescription opioid. Only one patient (2.4%) was prescribed a naloxone rescue kit upon discharge. There were two (4.8%) non-fatal overdoses within 3 months of admission. Thirteen PWID (31%) received SUD referrals and 12 (28.6%) PWID received psychiatry referrals upon discharge. Ninety- day mortality among PWID who were prescribed MOUD upon discharge was notably lower (3% vs 15%, p = 0.45), as were ED visits (10% vs. 41%, p = 0.41) compared to those who did not receive MOUD. (**Table 3)**.

**Table 3:** Substance use disorder characteristics among PWID with IDU-associated infective endocarditis.

Variable	IDU-IE
	n = 42
	n(%)
**Prior to Admission**	
Treated with MOUD	5 (12%)
Methadone	1 (2%)
Buprenorphine/naloxone	2 (5%)
Buprenorphine	2 (5%)
**Upon Discharge**	
Treated with MOUD^a^	10 (24%)
Methadone	5 (12%)
Buprenorphine/naloxone	5 (12%)
Prescribed opioid	16 (38%)
Prescribed naloxone	1 (2%)
Non-fatal overdoses, within 3 months	2 (5%)
SUD referral	13 (31%)
Psychiatry referral	12 (29%)
Completed IV antibiotics if discharged home with PICC line^b^	
Yes	6 (60%)
No	2 (20%)
Unknown	2 (20%)
ED visit by MOUD^a^	
Treated with MOUD upon discharge	4 (10%)
Not treated with MOUD upon discharge	16 (41%)
90 day mortality by MOUD^a^	
Treated with MOUD upon discharge	1 (3%)
Not treated with MOUD upon discharge	6 (15%)

Data reported in n (%) unless otherwise indicated

Abbreviations: PWID, people who inject drugs; IDU-IE, injection drug use-associated infective endocarditis; MOUD, Medication for Opioid Use Disorder; SUD, Substance Use Disorder; IV, intravenous; PICC, peripherally inserted central catheter

^a^ Data missing for n = 3 PWID

^b^ n = 10

Notably, among PWID, 52.4% were not immune to HBV and only 7.1% received a HBV vaccine during admission. Approximately 79% of patients were not tested for a sexually transmitted infection (STI) during their hospitalization.

### Multivariable analyses

Injection drug use was not associated with having an ED visit after discharge (adjusted odds ratio (AOR) 0.73, 95% CI 0.18–3.36) controlling for age, gender, CCI score, hepatitis C, and homelessness. Injection drug use was not associated with cardiac surgery (AOR 0.22, 95% CI 0.09–1.76) controlling for age, gender, comorbidity index score, prosthetic valve endocarditis and ICU stay. ICU stay was associated with cardiac surgery (AOR 14.8, 95% CI 3.00–72.60), whereas CCI score of > 3 (AOR 0.15, 95% CI 0.03–0.69) was less likely to be associated with cardiac surgery. Finally, injection drug use was not associated with mortality (AOR 0.72, 95% CI 0.17–3.01) after controlling for age, gender and higher CCI score. Patients with cardiac devices may have a higher risk for infective endocarditis; however, our results were materially unchanged when we excluded patients with cardiac devices in our sensitivity analyses.

## Discussion

In this study, there were several health differences between people with IDU-associated versus non-IDU infective endocarditis, and services for PWID were lacking. While injection drug use was not associated with cardiac surgery, morbidity or mortality, people with IDU-associated infective endocarditis had less comorbidities and were significantly younger than those with non-IDU infective endocarditis and yet still had similar rates of cardiac surgery, ED visits, and in-hospital mortality. The results of this study highlight the importance of addressing social determinants of health and delivering comprehensive harm reduction services and treatment among PWID.

Similar to prior literature, people with IDU-associated endocarditis in our study were more likely to be younger, reflecting the current trends of the opioid epidemic.[4, 15, 16] Interestingly, while the proportion of right vs. left sided infective endocarditis was higher among the IDU population, there were only a few cases of right-sided infective endocarditis overall in our study population. While IDU-associated endocarditis has most often been associated with right-sided valve disease, our study adds to the growing body of literature describing more left-sided disease in this population.[17] Notably, people with IDU-associated infective endocarditis had fewer comorbidities compared to those with non-IDU infective endocarditis, except for HCV, which was prevalent in 75% of PWID. The high prevalence of HCV among people with IDU-associated infective endocarditis emphasizes the need for not only screening of viral hepatitis, in this population, but also linkage to outpatient HCV treatment following the infective endocarditis admission.[18] In our study, admissions were a missed opportunity for STI screening as well as vaccinations, particularly for hepatitis B. Given the acute hepatitis B cases in Maine and rise of STIs nationally, vaccinating and screening of other STIs in at-risk hospitalized individuals should be a priority[19, 20]. In addition, the rising number of infective endocarditis cases among PWID observed in our study (12 cases in 2013 to 18 cases in 2015), suggests there are increased opportunities for health service optimization.

Other social determinants of health differed in statistical significance between the two groups. Consistent with prior literature, study participants with a history of injection drug use had a high prevalence of homelessness [21, 22] and mental health conditions [21], and were less likely to have health insurance [23]. As such, discharge disposition of people with IDU-associated infective endocarditis can be challenging. Notably, in our study 60% of people with IDU-associated endocarditis discharged with home IV antibiotics (n = 7) completed their treatment; most of these participants were housed, insured, and/or were discharged on MOUD. At our institution, discharge plans are made on a case-by-case basis in conjunction with expert opinion primarily from the addiction consult, infectious diseases, and hospitalist medicine teams. The success rate of people with IDU-associated infective endocarditis completing their treatment with home IV antibiotics, albeit a small number of select patients in this study, suggests that further research is needed on different medical models of outpatient antibiotic therapy in this population. Thus far, the use of a risk assessment tool to guide discharge decisions has been shown to help decreased length of stays and optimize resources for individuals with ongoing IDU that may remain hospitalized [24]. While a recent study suggested noninferiority of oral antibiotics compared with intravenous antibiotics for treatment of left sided infective endocarditis, PWID were vastly underrepresented (1.3% compared to 39% in our study population) [25]. In addition to discharging patients on oral versus parenteral antibiotics, more research is needed on the effectiveness of patient-directed and harm-reduction based discharges in individuals with IDU history and similar social determinants of health to our study population.

We found injection drug use was not associated with cardiac surgery, ED visits, or mortality in this study. Though not statistically significant, people with IDU-associated infective endocarditis had a slightly higher prevalence of cardiac surgery (33% vs. 26%). Participants with IDU-associated infective endocarditis had larger vegetations, infectious complications, and lack of comorbidities, and ICU stays. National guidelines do recommend early valve surgery for infective endocarditis in patients with serious complications, such as severe valve regurgitation, heart block, etc, thus these results were not surprising. [12]. Also, IDU was not associated with ED visits in this study; female gender, however, was predictive of ED utilization, which has also been shown in previous research [26, 27]. Finally, IDU was not associated with short-term mortality in this study; there was a non-statistically significant higher proportion of deaths among those with non-IDU infective endocarditis, possibly explained by more comorbidities in that group. Other studies have shown mixed results regarding the association between mortality and IDU. Injection drug use has been associated with lower operative risk, but increased risk of re-infection and valve complications, though data on whether or not patients in those studies were offered substance use disorder treatment following valve replacement are lacking. Prior literature has shown no difference in short-term mortality among people with IDU-associated infective endocarditis versus non-IDU infective endocarditis, though differences in long-term mortality have been described [28].

There were no statistically significant differences in the pathogens implicated in infective endocarditis between groups, but there was an increase in *Staphylococcus aureus* infections among people with IDU-associated infective endocarditis (54.8% compared to 38.5%). Our findings were consistent with other studies documenting *S*. *aureus* as the most common cause of IDU-associated infective endocarditis [29, 30]. Our institution has a policy that encourages infectious diseases consultations on patients with certain bacteremias; bedside infectious diseases consultations were performed in 98.1% of our study population. This has been previously described as an essential component in the management of *S*. *aureus* bacteremia. Several studies have found significantly better clinical outcomes, including decreased mortality and relapse, when an infectious diseases specialist provides bedside consultation [31, 32, 33, 34]. The high involvement of infectious diseases in our study may have lowered the number of morbidity and mortality events making it more difficult to detect a difference between people with IDU-associated infective endocarditis versus people with non-IDU infective endocarditis.

Recent data suggest that current substance use disorder interventions, particularly for individuals hospitalized with IDU-associated infective endocarditis, are suboptimal [17, 35]. Only a small proportion of PWID in this study were discharged on MOUD, likely due to lack of access to SUD care in a rural state and/or lack of insurance, as Maine did not participate in Medicaid expansion until after study completion. Notably, the state has since seen improvement in access to MOUD. [36], Only one participant was discharged with a naloxone rescue kit and 38.1% were discharged on a prescription opioid. Subsequent to this, opioid prescribing laws were developed in our state; nevertheless, the proportion of opioids prescribed on discharge was concerning. While our study was not powered to examine the effect of MOUD on mortality or morbidity, notably participants with IDU-associated infective endocarditis treated with MOUD had less ED visits and lower 90-day mortality rates compared to those not treated with MOUD. The impact of MOUD on IDU-associated infective endocarditis outcomes warrants further investigation. These findings also highlight the need to improve access to MOUD and other services among hospitalized PWID with infective endocarditis. The prevalence of co-occurring alcohol use and stimulant use among PWID also emphasizes the need to address potential co-occurring substance use disorders in this population. Integrating addiction medicine consultations has been associated with increased treatment for opioid use disorders and better outcomes in patients with IDU-associated infective endocarditis.[37] After the completion of this study, our institution has since implemented an inpatient addiction consult team as well as an electronic order to set to streamline screening for STIs and ordering of vaccines and naloxone rescue kits [18]. Given the increasing incidence of overdoses, particularly among people who report polysubstance use, such as our study population, naloxone prescribing is particularly important upon discharge [38, 39]. In patients who may not be candidates for MOUD, counseling around safe injection techniques should be employed, particularly to avoid not only infections such as infective endocarditis, but other infectious complications such as septic arthritis, osteomyelitis, epidural abscesses, and viral hepatitis which are also on the rise nationally[40].

## Limitations

Our study was a single-site study performed in a large, tertiary facility; therefore, results may not be generalizable to other institutions with different patient demographics. Notably, however, the study site is a large referral center that serves both urban and rural patients. In addition, patients were screened for inclusion into the study by TEE results,. While most patients who are suspected of having endocarditis undergo TEE at our institution, it is certainly possible that we have missed a few eligible patients who received TTE's alone. Therefore it remains possible that endocarditis cases were slightly underreported during the study period. Due to the retrospective nature of our study, we also were unable to specify a date range for IDU; rather, we defined IDU as any history of injection drug use. In addition, while we captured vascular phenomena, we did not differentiate between intracranial hemorrhage versus other types of vascular phenomena in our data collection. It is possible that the presence of ICH could have been a potential confounder for cardiac surgery, as some surgeons may not operate on those with ICH due to increased bleeding risk/concern for stroke exacerbation. Also, although IDU-associated infective endocarditis group consisted of 39% of the study size, our results may have been affected by our small sample size of 107 patients. As it was not significant in our unadjusted odds (bivariate) analyses, potentially due to small sample size, we also did not include valve affected in our multivariable analyses. Mortality data were limited to medical examiner and EHR data, so deaths may be underestimated. Only short-term mortality was assessed, and it would be useful to conduct a similar study with long-term mortality beyond 90 days. While we had access to a statewide database to capture ED visits, access to outpatient data were limited to our health system.

## Conclusions

Our results highlight existing differences in health characteristics and social determinants of health in people with IDU-associated versus non-IDU infective endocarditis. Moreover, people with IDU-associated infective endocarditis had less comorbidities and were significantly younger than those with non-IDU infective endocarditis and yet still had similar rates of cardiac surgery, ED visits, and in-hospital mortality. These findings emphasize the need to deliver comprehensive health services, particularly MOUD and other harm reduction services, to this marginalized population.

## Abbreviations

PWID: people who inject drugs
IDU: injection drug use
MOUD: Medication for Opioid Use Disorder
SUD: Substance Use Disorder
CT: cardiothoracic
IV: intravenous
PICC: peripherally inserted central catheter
ICU: intensive care
ED: emergency department
ID: infectious diseases
STI: sexually transmitted infection
HCV: hepatitis C virus
HBV: hepatitis B virus
HIV: human immunodeficiency virus
CCI: charlson comorbidity index
EHR: electronic health record
